# Unveiling the Riddoch phenomenon: a regression analysis of stroke-induced homonymous hemianopia

**DOI:** 10.3389/fneur.2025.1620349

**Published:** 2025-08-25

**Authors:** Oranan Tritanon, Suphakarn Kimavaha, Sukanya Siriyotha, Gordon T. Plant, Panitha Jindahra

**Affiliations:** ^1^Department of Diagnostic and Therapeutic Radiology, Faculty of Medicine, Ramathibodi Hospital, Mahidol University, Bangkok, Thailand; ^2^Division of Neurology, Department of Medicine, Faculty of Medicine, Ramathibodi Hospital, Mahidol University, Bangkok, Thailand; ^3^Biostatistics Department of Clinical Epidemiology and Biostatistics, Faculty of Medicine Ramathibodi Hospital, Mahidol University, Bangkok, Thailand; ^4^Department of Brain Repair and Rehabilitation, University College London, London, United Kingdom

**Keywords:** Riddoch phenomenon, V5, motion perception, homonymous hemianopia, Rama motion perimetry, blindsight, neural plasticity, diabetes mellitus

## Abstract

**Introduction:**

A subset of patients with homonymous hemianopia can consciously perceive motion within their blind visual fields—a phenomenon known as the Riddoch phenomenon. However, the factors predicting this residual motion perception remain poorly understood. This study aims to identify clinical and neuroanatomical predictors of the Riddoch phenomenon in stroke patients.

**Methods:**

We retrospectively analyzed 32 adult patients (mean age 60.41 years, 34.4% female) with stroke-induced homonymous hemianopia treated at a single center between 2020 and 2023. Clinical data, brain MRI, and visual field assessments were reviewed. The Riddoch phenomenon was quantified as the difference between kinetic motion perception measured by Rama Motion Perimetry (RMP) and static visual perception assessed by Humphrey Visual Field Index (VFI), termed RMP-VFI. Lesions in key visual processing regions—primary visual cortex (V1), motion-sensitive area V5, lateral geniculate nucleus (LGN), and splenium of the corpus callosum—were identified on MRI. Univariate and multiple linear regression models were applied to evaluate predictors of RMP-VFI.

**Results:**

Mean RMP-VFI scores were significantly higher in patients with spared V5 compared to those with lesioned V5 (24.1 vs. 1.8, *p* = 0.033), while no significant differences were observed for other regions. Multiple linear regression revealed diabetes mellitus as a significant negative predictor of RMP-VFI (*β* = −24.6, 95% CI: −44.47, −4.75; *p* = 0.017), with spared V5 showing a positive but marginally non-significant association (*β* = 17.5, 95% CI: −1.61, 36.66; *p* = 0.071). The model explained 30% of the variance in RMP-VFI (adjusted R^2^ = 0.25).

**Discussion:**

Integrity of area V5 plays a key role in the Riddoch phenomenon by preserving motion perception despite cortical damage. For the first time, diabetes mellitus is identified as a significant clinical factor negatively influencing residual motion perception, possibly by impairing neural plasticity. These findings enhance understanding of the neural and systemic factors modulating visual recovery after stroke.

## 1 Introduction

Homonymous hemianopia (HH) is a neurological condition in which patients cannot perceive visual stimuli on the same side of their visual field in both eyes. This condition results from a lesion or lesions located posterior to the optic chiasm, most commonly along the geniculo-striate pathway, which consists of fibers that project from the lateral geniculate nucleus (LGN) to the primary visual cortex (V1). The geniculo-striate pathway has a retinotopic organization, meaning that specific regions of the pathway correspond to specific visual field areas. Injuries to these fibers results in cortical blindness in the corresponding region of the visual field (1–4).

Certain individuals diagnosed with HH or cortical blindness exhibit residual vision, allowing them to detect movement, pattern, or color within their blind hemifields (5–8). The extent and characteristics of this residual vision can differ significantly among patients.

The Riddoch Phenomenon is a distinctive category of residual vision observed in individuals with complete or partial HH or cortical blindness (bilateral complete HH). These individuals can consciously perceive movement in their blind hemifield (5). This phenomenon was first described by Riddoch in 1917, and it was later named Riddoch syndrome by Zeki and Ffytche (9). In Riddoch’s observations, patients cannot perceive stationary objects in the blind hemifield. Yet, they could quickly recognize something moving when the objects oscillated—a phenomenon called stato-kinetic dissociation. The “something moving” appears vague or shadowy, lacking distinct shapes and colors (5). A study of one such patient found that their contrast sensitivity to temporally modulated stimuli was within normal limits (10). While the pathology in most of the cases described has been destructive lesions, mostly stroke, the Riddoch phenomenon is a very common finding in the degenerative disorder Posterior Cortical Atrophy, which is most commonly associated with Alzheimer’s pathology (11).

In 1974, Weiskrantz et al. described the term blindsight in a 26-year-old man following V1 resection (12, 13). Psychophysical studies revealed that he was able to detect and point to moving stimuli in the blind hemifield above chance. He was unaware of seeing the stimuli in the blind hemifield and reported that he was guessing. His experience differs from the Riddoch phenomenon in the level of conscious awareness associated with perception but also in the detection threshold which is an order of magnitude higher (less sensitive) than that found in the Riddoch phenomenon (10). Thus, it can be understood that the perception of movement in the blind area ranges from subconscious to conscious awareness (14–18). Furthermore, blindsight has been observed even in cases of complete loss of the whole cerebral hemisphere following hemispherectomy, which rules out the possibility of the preserved motion perception being due to spared islands of V1 (19) or indeed involvement of other ipsilesional cortical areas such as the motion area V5.

It is essential to highlight that the visual field defect and residual vision are relevant only under the specific conditions being tested (6). For instance, HH is, following the introduction of automated perimetry, usually diagnosed through static testing, while residual visual motion perception is assessed using kinetic perimetry. The exploration of the borderland between conscious and unconscious perception of visual stimuli following damage to the occipital cortex is of considerable interest.

Based on animal research, there have been suggestions that there is a pathway directly from the retina to the midbrain, separately from the geniculo-cortical route. Recent advancements in research using functional magnetic resonance imaging (fMRI) and diffusion tensor imaging (DTI) have provided important insights into the mechanisms behind conscious and unconscious preserved motion perception. One significant fMRI study investigated the neurobiology of the Riddoch phenomenon in a patient named GY, who developed HH due to a lesion in area V1 of the visual cortex. The results showed that V5 consistently exhibited activity when moving stimuli were presented in the blind hemifield that the subject experienced (20). Additionally, another study utilized a positron emission tomography scan on GY, which revealed that V5 was active even in the absence of activity in V1 (21). This indicates direct visual input to V5.

Growing evidence from DTI suggests that a direct pathway from the LGN to V5 is responsible for the preserved motion perception in HH. This bypasses the geniculo-striate pathway from the LGN to V1 (20–25). However, previous studies have revealed a role of the superior colliculus in the unconscious preserved motion perception in hemispherectomy patients (15).

A recent study by DTI quantified the Riddoch phenomenon by measuring the difference between the Rama motion perimetry (RMP) scores (Kinetic stimuli) and the visual field index (VFI) from Humphrey perimetry (static stimuli), referred to as RMP-VFI (26). This measurement reflects the fiber connectivity density between the contralesional LGN - contralesional V5 and between contralesional V5—contralesional LGN in patients with HH. Additionally, the fiber connectivity density between the contralesional LGN—contralesional V5 increased after unilateral visual pathway damage. Therefore, RMP-VFI may serve as an indicator of neural plasticity involving the contralesional hemisphere (26).

Not all patients with HH experience the Riddoch phenomenon, and our understanding of the factors that predict its occurrence is still limited. It is still unclear whether the spared V5 and other areas in structural magnetic resonance imaging (MRI) would be associated with the phenomenon, or whether any underlying diseases, such as diabetes, can influence the Riddoch phenomenon. This study aims to determine, among adult stroke patients with HH, whether the presence of spared motion-sensitive visual cortex (V5) and clinical factors such as diabetes mellitus compared to lesioned V5 and absence of diabetes are associated with greater residual conscious motion perception quantified by the difference between RMP and VFI.

## 2 Materials and methods

This retrospective study was conducted in the Division of Neurology within the Department of Medicine at Ramathibodi Hospital, Mahidol University, from January 2020 to December 2023. The institutional ethics committee approved the study protocol. Additionally, this study did not utilize any identifiable biospecimens. The investigators did not have direct contact with the participants in the study. This study presented minimal risk and met Food and Drug Administration criteria for waiver of all consents.

### 2.1 Participants

Patients who were listed in an electronic database were included in the study. Adult patients with homonymous visual field defects were eligible for this study if they met the following criteria: age ≥ 18 years old; having lesions that cause homonymous visual field defects, such as HH or cortical blindness at the posterior visual pathway such as the lateral geniculate nucleus, occipital lobe, and optic radiation; visual field perimetric studies were available from routine clinical assessments; the visual field index was less than 60; brain MRI was available from routine clinical assessments. Patients were excluded if they had lesions at the anterior visual pathway, such as retina, glaucoma, and optic neuropathy. Demographic and clinical data were recorded from our electronic medical records, including age, sex, atrial fibrillation, diabetes mellitus, hypertension, dyslipidemia, chronic kidney disease, duration, disease, and the Trial of Org 10,172 in Acute Stroke Treatment (TOAST) classification of ischemic stroke (large-artery atherosclerosis, cardioembolic, small vessel occlusion, stroke of other determined etiology, and stroke of undetermined etiology) (27).

### 2.2 Perimetry and Riddoch phenomenon evaluation

Static Perimetry was available from routine clinical assessments, Humphrey Perimetry (HFA). All patients underwent HFA (24–2, SITA-Standard, Humphrey Systems, Dublin, CA, United States) at the initial presentation. The HFA projects a series of static white light spots with varying light intensities over five orders of magnitude, from 10,000 apostilbs (asb) to 0.1 asb at pre-determined locations in the bowl. The stimulus size is 4 mm2, and the duration 0.2 s. Testing is monocular, and appropriate lens correction is provided. Patients fixate on a central target responding with a button press when a target is perceived. The VFI is the outcome of the test. VFI quantifies performance, ranging from 100% correct to total loss at 0%. The mean VFI of the two eyes was calculated. The VFI represents the percentage of visual function of the entire visual field. It is derived from the age-corrected defect depth at the test points and was significantly depressed in the pattern deviation probability maps. The VFI ranges from 0 (blindness) to 100% (normal vision) (28). HFA is a standard perimetric technique for measuring conscious perception of statically presented stimuli. Thus, a patient with complete HH will have a VFI of approximately 50%. It should be noted that the patients were instructed to press whenever a stimulus was seen and were not encouraged to guess: the methodology will not, therefore, demonstrate subconscious blindsight. Also, the methodology tests out to 24° of visual angle from fixation. It does not test the entire visual hemifield (the standard visual field measures 90° temporally to central fixation, 50 degrees superiorly and nasally, and 60 degrees inferiorly). Therefore, residual vision at the extreme periphery will not be detected.

Kinetic perimetry was performed using RMP. It is a web-based perimetry measured in a room lit by an electric bulb. The visual stimuli are small black dots (negative contrast, luminance 0 cd/m2) moving clockwise with a 6°/ms velocity. The dots appear randomly in time and location on a computer screen (luminance 1 cd/m2) within the central 24° of the visual field. The viewing distance is 30 cm. Patients were instructed to fixate on a central target binocularly and to click a spacebar as soon as they perceived any movement regardless of form perception. The result is expressed as a percentage correct. RMP is considered valid when errors (false negative and false positive) detected in the program are close to zero.

The difference between %correct in RMP and %correct VFI in HFA (RMP-VFI) indicates the level of preserved conscious motion perception or degree of stato-kinetic dissociation (i.e., the Riddoch phenomenon). The greater the difference, the greater the level of residual motion perception. We only included patients with a VFI of less than 60 because the Riddoch phenomenon typically occurs in cases with larger blind areas. Patients with more minor visual field defects who can see most of their visual field rarely experience the Riddoch phenomenon. HH among the patients included in the study presented in various forms: complete, incomplete, congruous, and incongruous.

### 2.3 Brain magnetic resonance imaging

All patients underwent brain MRI using a 3.0 T MRI scanner (Ingenia; Philips Healthcare, Best, the Netherlands). The MRI protocols included axial and coronal T1-weighted images, T2-weighted images, fluid-attenuated inversion recovery (FLAIR), diffusion-weighted imaging (DWI), and apparent diffusion coefficients (ADC). Regions of interest (ROIs) included bilateral V1, bilateral V5, bilateral LGN, and the splenium of the corpus callosum. All MRI scans underwent quality control and visual inspection. Scans with excessive motion artifacts, incomplete brain coverage, or low signal-to-noise ratio were excluded. ROIs were determined in a template scan prior to the review to ensure consistency and reduce inter-scan variability. One of the investigators (OT) reviewed the MRI. ROIs with hypersignal lesions on FLAIR and/or restricted diffusion on DWI and ADC were considered abnormal. The lesioned ROIs had at least one lesion in one hemisphere, while the spared ROIs had no lesion in either hemisphere.

### 2.4 Statistical analysis

Data were presented using either the mean and standard deviation (SD) or the median and range, depending on the appropriateness of continuous variables, while percentages were used for categorical variables. The normality of the data was assessed using the Shapiro–Wilk test. The results showed no significant deviation from a normal distribution (W = 0.965, *p* = 0.372). Hence, the assumption of normality is satisfied, allowing for the appropriate application of parametric and regression analyses. Comparisons of RMP-VFI between lesioned and spared regions of interest were analyzed using an independent samples T-test. The relationships between RMP-VFI and potential predictors of the Riddoch phenomenon were evaluated through univariate linear regression, with estimated coefficients and a 95% confidence interval (CI). Variables that demonstrated statistical significance (*p* < 0.05) during the univariate analysis were included as covariates in the multiple linear regression model to assess the combined effect of selected variables and to test for potential confounders. To ensure model adequacy, the model was evaluated using adjusted R^2^, variance inflation factors (VIF), and residual plots. We ensured that the assumptions of linearity, independence, homoscedasticity, and normality of residuals were met. Statistical analyses were conducted using STATA version 18 (Stata Corp., College Station, Texas, United States), with a *p*-value of less than 0.05 deemed statistically significant. The required sample size was determined based on a power analysis conducted prior to data collection (26). Assuming a large effect size (f^2^ = 0.35) and a significance level (*α*) of 0.05, a total sample size of at least 31 was needed to achieve a power of 0.80 (80%), ensuring an adequate probability of detecting a statistically significant effect if the null hypothesis was false.

## 3 Results

A total of 32 patients with homonymous visual field defects resulting from stroke were included in the analysis. Table 1 summarizes the demographic and clinical characteristics of the cohort. The sample comprised 11 females (34.4%) and 21 males (65.6%), with a mean age of 60.41 years (SD = 15.52). The median duration of the disease was 171.5 days, ranging from 0 to 7,075 days. Of the participants, 30 (94%) presented with cerebral infarction, while 2 (6%) had intracerebral hemorrhage.

**Table 1:** Demographic and clinical data.

Characteristics	*N* = 32
Age, years, mean (SD)	60.41 (15.52)
Sex, n (%)	
Female	11 (34.4)
Male	21 (65.6)
Atrial fibrillation, n (%)	7 (21.9)
Diabetes mellitus, n (%)	8 (25.0)
Duration in years, median (range)	5.5 (1–10)
HbA1C, median (range)	5.58 (4.49, 8.67)
Type 2 diabetes mellitus, %	100%
Hypertension, n (%)	16 (50.0)
Dyslipidemia, n (%)	14 (43.8)
Chronic kidney disease, n (%)	7 (21.9)
TOAST, n (%)	
1 Large-artery atherosclerosis	8 (26.7)
2 Cardioembolic	8 (26.7)
3 Small vessel occlusion	3 (10.0)
4 Stroke of other determined etiology	7 (23.3)
5 Stroke of undetermined etiology	4 (13.3)
Regions of interest in magnetic resonance imaging	
V1, n (%)	
Lesioned	27 (84.4)
Spared	5 (15.6)
V5, n (%)	
Lesioned	23 (71.9)
Spared	9 (28.1)
Lateral geniculate nucleus, n (%)	
Lesioned	18 (56.3)
Spared	14 (43.7)
Splenium of the corpus callosum, n (%)	
Lesioned	19 (59.4)
Spared	13 (40.6)
Rama motion perimetry	
Total % correct, mean (SD)	51.52 (30.48)
Total % correct, median (range)	57.35 (1.47, 98.53)
Visual field index	
Mean (SD)	42.75 (15.76)
Median (range)	48.25 (1.50, 59.50)
Riddoch phenomenon (RMP-VFI)	
Mean (SD)	8.07 (26.91)
Median (range)	9.78 (−54.59, 47.53)
Duration, median (range)	171.5 (0, 7,075)

SD, standard deviation; TOAST, the Trial of Org 10,172 in Acute Stroke Treatment; RMP, Rama motion perimetry; VFI, Visual field index.

The mean RMP score was 51.52 (SD = 30.48), and the mean VFI was 42.75 (SD = 15.76). The mean difference between RMP and VFI, which corresponds to the Riddoch phenomenon, was 8.07 (SD = 26.91).

An independent samples t-test revealed that the mean RMP-VFI was significantly greater in the spared V5 group (mean = 24.09, SD = 21.18) compared to the lesioned V5 group (mean = 1.81, SD = 26.68; *p* = 0.033). No statistically significant differences were identified in mean RMP-VFI between spared and lesioned groups for other regions of interest (Table 2).

**Table 2:** Comparisons between RMP-VFI and regions of interest using a T-test.

Regions of interest in magnetic resonance imaging	Mean (SD)	Mean difference (95% CI)	*p*-value
V1			
Spared	21.50 (16.51)	15.92 (42.47, −10.63)	0.230
Lesioned	5.59 (27.94)	0	
V5			
Spared	24.09 (21.18)	22.28 (42.62, −1.95)	0.033*
Lesioned	1.81 (26.68)	0	
Lateral geniculate nucleus			
Spared	12.89 (28.44)	8.56 (28.21, −11.10)	0.381
Lesioned	4.33 (25.85)	0	
Splenium of the corpus callosum			
Spared	10.25 (28.03)	3.68 (23.74, −16.39)	0.711
Lesioned	6.58 (26.79)	0	

RMP-VFI, the differences between visual field index and Rama motion Perimetry; SD, standard deviation; CI, confidence interval; *statistically significant.

A univariate linear regression analysis was performed to identify potential predictors of RMP-VFI (Table 3). Two covariates, diabetes mellitus and spared V5, demonstrated notable coefficients, with *p*-values below the threshold of 0.05. Multiple linear regression revealed diabetes mellitus as a significant negative predictor of RMP-VFI (*β* = −24.6, 95% CI: −44.47, −4.75; *p* = 0.017), with spared V5 showing a positive but marginally non-significant association (*β* = 17.5, 95% CI: −1.61, 36.66; *p* = 0.071). The model explained 30% of the variance in RMP-VFI (adjusted R^2^ = 0.25; Table 4). The Variance Inflation Factor (VIF) for diabetes mellitus and spared V5 was calculated to be 1.04 for each variable. These values suggest minimal multicollinearity, indicating that both predictors correlate poorly with other explanatory variables within the regression model. Such VIF values are well below commonly cited thresholds (e.g., 5 or 10), affirming the stability and reliability of the coefficient estimates associated with these predictors. A residual plot (homoscedasticity check) displayed a random scattered fashion, indicating that the linear regression model is appropriate for the data.

**Table 3:** Factors associated with the differences between Rama motion perimetry and visual field index (the Riddoch phenomenon): a univariate linear regression analysis.

Covariates	Coefficient (95%CI)	*p*-value
Age, years	0.01 (−0.63, 0.66)	0.963
Sex		
Male	−8.34 (−28.9, 12.22)	0.414
Female	0	
Atrial fibrillation		
Yes	3.81 (−20.04, 27.66)	0.746
No	0	
Diabetes mellitus		
Yes	−28.27 (−48.49, −8.04)	0.008*
No	0	
Diabetes mellitus		
Duration, years	−5.00 (−31.15, 21.15)	0.686
HbA1C, %	1.64 (-72.76, 76.04)	0.963
Hypertension		
Yes	−6.60 (−26.20, 13.00)	0.497
No	0	
Dyslipidemia		
Yes	−7.17 (−26.90, 12.56)	0.464
No	0	
Chronic kidney disease		
Yes	−13.02 (−36.42, 10.37)	0.265
No	0	
TOAST		
1. Large-artery atherosclerosis	0	
2. Cardioembolic	−5.38 (−34.34, 23.58)	0.705
3. Small vessel occlusion	−4.35 (−43.56, 34.87)	0.821
4. Stroke of other determined etiology	−11.86 (−41.84, 18.11)	0.423
5. Stroke of undetermined etiology	−10.37 (−45.84, 25.10)	0.553
Duration	0.0024 (−0.0026, 0.0115)	0.209
Regions of interest in MRI		
V1		
Spared	15.92 (−10.63, 42.47)	0.230
Lesioned	0	
V5		
Spared	22.28 (1.95, 42.62)	0.033*
Lesioned	0	
Lateral geniculate nucleus		
Spared	8.56 (−11.09, 28.21)	0.381
Lesioned	0	
Splenium of the corpus callosum		
Spared	3.68 (−16.39, 23.74)	0.711
Lesioned	0	

CI, confidence interval; TOAST, the Trial of Org 10,172 in Acute Stroke Treatment; MRI, magnetic resonance imaging; *statistically significant.

**Table 4:** Factors associated with the differences between Rama motion perimetry and visual field index (RMP-VFI or the Riddoch phenomenon): a multiple linear regression analysis [RMP-VFI = 9.30–24.61(Diabetes mellitus) + 17.53(Spared V5); R^2^ = 0.3, adjusted R^2^ = 0.25].

Covariates	Coefficient (95%CI)	*p*-value
Diabetes mellitus		
Yes	−24.61 (−44.47, −4.75)	0.017*
No	0	
V5		
Spared	17.53 (−1.61, 36.66)	0.071
Lesioned	0	

CI, confidence interval; MRI, magnetic resonance imaging; *statistically significant.

## 4 Discussion

The results showed that the mean RMP-VFI was significantly greater in the spared V5 group than in the lesioned V5 group. Our findings from structural brain MRI align with those observed in functional MRI and DTI studies (20–25). Previous research has indicated that the connection between the LGN and V5 is crucial for preserved motion perception following damage to occipital cortex. This study provides additional information about how the Riddoch phenomenon in patients with HH due to stroke in the geniculo-striate pathway can assist in neuroanatomical localization on brain MRI. The RMP-VFI methodology has the additional advantage that relative preservation of detection of moving stimuli regionally within the visual field can be studied in addition to the extreme case of total loss of detection of static stimuli with preservation of motion perception.

The multiple linear regression analysis revealed that diabetes mellitus (*p* = 0.017) was identified as a statistically significant predictor of RMP-VFI, while spared V5 (0.071) did not achieve statistical significance. Individuals with diabetes have an RMP-VFI that is 24.6 units lower than those without diabetes, after controlling for other factors. The analysis highlights that diabetes is significantly associated with RMP-VFI (the Riddoch phenomenon). However, due to the retrospective nature of the study design, this finding reflects an association—not a direct causal relationship—between diabetes and RMP-VFI. Spared V5, despite failing to achieve statistical significance, suggests a potential relationship and a trend. This likely reflects limited statistical power due to the modest sample size and suggests that a larger cohort would be beneficial to confirm these findings. Additionally, the adjusted R^2^ value reflects moderate explanatory power, though additional covariates might improve the model’s fit.

According to Sungkarat et al., RMP-VFI acts as a marker for neural plasticity, specifically highlighting how the brain adapts to preserve motion perception despite damage to visual pathways (26). The fiber connectivity density between the LGN and V5 in the unaffected hemisphere increases when the ipsilateral visual pathway is injured in patients with HH—this change results in preserved motion perception within the blind area. Our results imply that diabetes may impair neural plasticity mechanisms. The findings could influence patient management, prognosis, or rehabilitation strategies. In particular, diabetic patients might benefit less from rehabilitation targeting motion perception or neural plasticity. This lays the groundwork for further exploration in the future.

There is prior evidence in favor of this interpretation: specifically the effect of diabetes mellitus on neural plasticity: Chronic hyperglycemia, a hallmark of diabetes, can alter synaptic plasticity in the brain, leading to cognitive deficits such as reduced mental speed and flexibility (29–31). Persistent facilitation of long-term depression and inhibition of long-term potentiation in the hippocampus may contribute to learning and memory impairments associated with diabetes mellitus (30). Diabetes-related oxidative stress and alterations in cerebral microvasculature can further impair neural plasticity. Compared with healthy control subjects, functional connectivity, and cognition differed in type 1 diabetic patients irrespective of microvascular complication status, indicating that chronic hyperglycemia may negatively affect brain functioning even before microvascular damage manifests (31). Advanced glycation end products generated by chronic hyperglycemia and their receptor RAGE provide critical links between diabetes and AD (32). The findings have the potential to deepen our understanding of the systemic impacts of diabetes on the brain’s adaptability.

The study has some limitations, which are outlined as follows. This study was retrospective and was conducted at a single center, which may introduce selection bias and limit the generalizability of the findings to other populations, stroke etiologies, or neuro-ophthalmological conditions. There was no functional MRI or diffusion tensor imaging to validate the neural substrates underlying the RMP-VFI. The limitations of RMP measurement include potential ceiling or floor effects of the test, as well as variability in patient cooperation during testing. A larger prospective study that combines multimodal imaging (both structural and functional) with detailed clinical phenotyping and more covariates is essential to validate and extend the current findings. Such studies would improve mechanistic understanding and potentially enhance clinical translation.

## 5 Conclusion

Integrity of area V5 plays a key role in the Riddoch phenomenon by preserving motion perception despite cortical damage. For the first time, diabetes mellitus is identified as a significant clinical factor negatively influencing residual motion perception, possibly by impairing neural plasticity. These findings enhance understanding of the neural and systemic factors modulating visual recovery after stroke.

## Data Availability

The raw data supporting the conclusions of this article will be made available by the authors, without undue reservation.

## References

[1] Mehta JS Plant GT. Optical coherence tomography (OCT) findings in congenital/long-standing homonymous hemianopia. Am J Ophthalmol. (2005) 140:727–9. doi: 10.1016/j.ajo.2005.03.059, PMID: 16226527

[2] Jindahra P Petrie A Plant GT. Retrograde trans-synaptic retinal ganglion cell loss identified by optical coherence tomography. Brain. (2009) 132:628–34. doi: 10.1093/brain/awp001, PMID: 19224900

[3] Bridge H Jindahra P Barbur J Plant GT. Imaging reveals optic tract degeneration in hemianopia. Invest Ophthalmol Vis Sci. (2011) 52:382–8. doi: 10.1167/iovs.10-5708, PMID: 20739474

[4] Koiava N Ong YH Brown MM Acheson J Plant GT Leff AP. A 'web app' for diagnosing hemianopia. J Neurol Neurosurg Psychiatry. (2012) 83:1222–4. doi: 10.1136/jnnp-2012-302270, PMID: 22645255

[5] Riddoch G. Dissociation of visual perceptions due to occipital injuries, with especial reference to appreciation of movement. Brain. (1917) 40:15–57.

[6] Bender MB Krieger HP. Visual function in perimetrically blind fields. AMA Arch Neurol Psychiatry. (1951) 65:72–9. 14782771 10.1001/archneurpsyc.1951.02320010078009

[7] Pöppel E Held R Frost D. Residual visual function after brain wounds involving the central visual pathways in man. Nature. (1973) 243:295–6. 4774871 10.1038/243295a0

[8] Perenin M Jeannerod M. Residual vision in cortically blind hemifields. Neuropsychologia. (1974) 13:1–7. 10.1016/0028-3932(75)90041-x 1109450

[9] Zeki S Ffytche DH. The Riddoch syndrome: insights into the neurobiology of conscious vision. Brain. (1998) 121:25–45. 9549486 10.1093/brain/121.1.25

[10] Plant GT Wilkins AJ. Preserved movement sensitivity following occipital lobe damage—a case report of spatio-temporal contrast sensitivity in the Riddoch phenomenon. Clin Vis Sci. (1988) 2:321–9.

[11] Maia da Silva M James-Galton M Green C Plant GT. Homonymous hemianopia in posterior cortical atrophy: right-left asymmetry, progression over time and relationship to the classical neuropsychological deficits. Brain Commun. (2025), 8:389. doi: 10.3389/fneur.2017.00389 PMC13008642 41884339

[12] Weiskrantz L Warrington EK Sanders MD Marshall J. Visual capacity in the hemianopic field following a restricted occipital ablation. Brain. (1974) 97:709–28. 4434190 10.1093/brain/97.1.709

[13] Sanders MD Warrington EK Marshall J Wieskrantz L. Blindsight: vision in a field defect. Lancet. (1974) 1:707–8. 4132425 10.1016/s0140-6736(74)92907-9

[14] Cowey A Stoerig P. Visual detection in monkeys with blindsight. Neuropsychologia. (1997) 35:929–39. 9226655 10.1016/s0028-3932(97)00021-3

[15] Weiskrantz L. Unconsciousness and commentaries In: Hameroff S Kaszniak A Scott A, editors. Towards a science of consciousness II—The second Tucson discussion and debates. Cambridge, MA: MIT Press (1989). 371–7.

[16] Weiskrantz L Barbur JL Sahraie A. Parameters affecting conscious versus unconscious visual discrimination with damage to the visual cortex (V1). Proc Natl Acad Sci USA. (1995) 92:6122–6. 7597090 10.1073/pnas.92.13.6122 PMC41654

[17] Barbur JL Ruddock KH Waterfield VA. Human visual responses in the absence of the geniculo-calcarine projection. Brain. (1980) 103:905–28. 7437894 10.1093/brain/103.4.905

[18] Danckert J Rossetti Y. Blindsight in action: what can the different subtypes of blindsight tell us about the control of visually guided actions? Neurosci Biobehav Rev. (2005) 29:1035–46. doi: 10.1016/j.neubiorev.2005.02.001, PMID: 16143169

[19] Ptito A Leh SE. Neural substrates of blindsight after hemispherectomy. Neuroscientist. (2007) 13:508–18. doi: 10.1177/1073858407300598 17901259

[20] Rees G. Neural correlates of the contents of visual awareness in humans. Philos Trans R Soc Lond Ser B Biol Sci. (2007) 362:877–86. doi: 10.1098/rstb.2007.2094, PMID: 17395576 PMC2430003

[21] Barbur JL Watson JD Frackowiak RS Zeki S. Conscious visual perception without V1. Brain. (1993) 116:1293–302. 8293272 10.1093/brain/116.6.1293

[22] Azzopardi P Cowey A. Motion discrimination in cortically blind patients. Brain. (2001) 124:30–46. doi: 10.1093/brain/124.1.30, PMID: 11133785

[23] Ajina S Kennard C Rees G Bridge H. Motion area V5/MT+ response to global motion in the absence of V1 resembles early visual cortex. Brain. (2015) 138:164–78. doi: 10.1093/brain/awu328, PMID: 25433915 PMC4285193

[24] Ajina S Bridge H. Blindsight relies on a functional connection between hMT+ and the lateral geniculate nucleus, not the pulvinar. PLoS Biol. (2018) 16:e2005769. doi: 10.1371/journal.pbio.2005769, PMID: 30044775 PMC6078309

[25] Ajina S Pestilli F Rokem A Kennard C Bridge H. Human blindsight is mediated by an intact geniculo-extrastriate pathway. eLife. (2015) 4:e08935. doi: 10.7554/eLife.08935, PMID: 26485034 PMC4641435

[26] Sungkarat W Chaeyklinthes T Thadanipon K Plant GT Jindahra P. Preserved motion perception and the density of cortical projections to V5 in homonymous hemianopia. Brain Commun. (2024) 6:fcaa436. doi: 10.1093/braincomms/fcae436, PMID: 39703325 PMC11656197

[27] Adams HP Jr Bendixen BH Kappelle LJ Biller J Love BB Gordon DL. Classification of subtype of acute ischemic stroke. Definitions for use in a multicenter clinical trial. TOAST. Stroke. (1993) 24:35–41. 7678184 10.1161/01.str.24.1.35

[28] Iutaka NA Grochowski RA Kasahara N. Correlation between visual field index and other functional and structural measures in Glaucoma patients and suspects. J Ophthalmic Vis Res. (2017) 12:53–7. doi: 10.4103/jovr.jovr_98_16, PMID: 28299007 PMC5340064

[29] Jones DT. Neural networks, cognition, and diabetes: what is the connection? Diabetes. (2012) 61:1653–5. doi: 10.2337/db12-0402, PMID: 22723270 PMC3379670

[30] Artola A Kamal A Ramakers GM Biessels GJ Gispen WH. Diabetes mellitus concomitantly facilitates the induction of long-term depression and inhibits that of long-term potentiation in hippocampus. Eur J Neurosci. (2005) 22:169–78. doi: 10.1111/j.1460-9568.2005.04205.x, PMID: 16029206

[31] van Duinkerken E Klein M Schoonenboom NS Hoogma RP Moll AC Snoek FJ. Functional brain connectivity and neurocognitive functioning in patients with long-standing type 1 diabetes with and without microvascular complications: a magnetoencephalography study. Diabetes. (2009) 58:2335–43. doi: 10.2337/db09-0425, PMID: 19584309 PMC2750229

[32] Pugazhenthi S Qin L Reddy PH. Common neurodegenerative pathways in obesity, diabetes, and Alzheimer's disease. Biochim Biophys Acta Mol basis Dis. (2017) 1863:1037–45. doi: 10.1016/j.bbadis.2016.04.017, PMID: 27156888 PMC5344771

